# Impact of a heterogeneous environment on the population expansion of the harmful plant *Iris ruthenica* Ker-Gawl. in the high mountain grasslands

**DOI:** 10.3389/fpls.2024.1363496

**Published:** 2024-05-03

**Authors:** Gulinige Tayier, Dilixiati Hasimu, Tayierjiang Aishan, Amanula Yimingniyazi

**Affiliations:** ^1^ Xinjiang Key Laboratory for Ecological Adaptation and Evolution of Extreme Environment Biology, College of Life Sciences, Xinjiang Agricultural University, Urumqi, Xinjiang, China; ^2^ College of Ecology and Environment, Xinjiang University, Urumqi, China

**Keywords:** slope direction, *Iris ruthenica*, flowering phenology, floral characteristics, breeding system, pollination characteristics

## Abstract

*Iris ruthenica* Ker-Gawl. (Russian iris) is a perennial, clonal, herbaceous plant that is spread across the degraded mountain grasslands in northern Xinjiang. In this study, to explore the breeding system and the impact of slope orientation on the flowering phenology, pollination characteristics, and other aspects of *I. ruthenica*, we used a combination of field observations and controlled experiments to compare the population density, flowering phenology, floral characteristics, breeding system, and pollinator type and behavior of plants on different slope orientations. Vegetation coverage on the north (N), southwest (SW), and southeast (SE) slopes was 90%, 67%, and 53%, respectively. Differences in plant height and diameter were observed between the SE slope and the N and SW slopes, whereas the N and SW slopes were similar in these respects. The SE slope exhibited the earliest initial flowering time, followed by the SW and N slopes. The diameter of the corolla, distance between the stigma and anther, length of the anther and ovary, number of pollen grains, and number of ovules on the N slope were smaller than those on the SE and SW slopes, whereas those of the SE and SW slopes were similar. Artificial pollination experiments showed that neither bagging nor unbagging resulted in seed formation after emasculation for all slope orientations. The pollinating insects of *I. ruthenica* included *Bombus* sp., *Amegilla leptocoma*, *Andrena* sp., and *Halictus* sp.; the types and numbers of pollinating insects differed among slopes. In summary, on SE and SW slopes with high temperatures and sufficient sunlight, this species attracted pollinators and provided them with more opportunities to visit and pollinate by flowering early, with large numbers of blooms, and a longer flowering period and lifespan, ensuring successful reproduction. Under unfavorable conditions, such as insufficient pollinators and limited activity caused by the more stressful environmental conditions of the N slope (including low temperature and insufficient light), this species ensures a certain seed yield by increasing its self-compatibility.

## 1 Introduction

High mountain ecosystems are characterized by large temperature differences between day and night, substantial changes in non-biological factors, and strong non-biological diversity. High mountain ecosystems not only have a strong impact on the adaptability and evolution of plants but also on the types and numbers of pollinators, thereby affecting the survival of high mountain plants in extreme environments (Peñuelas et al., 2004; Natsuki et al., 2022). For instance, adverse factors such as low temperatures, strong winds, heavy rain, and short growing seasons caused by changes in slope orientation and altitude can limit the types and activities of pollinating insects, resulting in a generally lower frequency of flower visits (Wang et al., 2020; Xiao et al., 2022), affecting the types of alpine plant breeding systems, and potentially leading to stronger pollen limitation for plants (Jacobi and del Sarto, 2007; López et al., 2021). Some alpine plants increase their flower lifespan and display area with changes in altitude and slope direction, and their reproductive allocation ratio compensates for the impact of limited pollinator activity on pollination efficiency, thereby improving the success of plant pollination (Buxton et al., 2016; Mao et al., 2019). Some alpine plants may adapt to harsh environmental conditions through wind pollination and asexual reproduction (Zhang et al., 2020). Alpine plants may evolve flower traits that favor outcrossing, thus attracting more pollinators and increasing fitness benefits by reducing the adverse effects of an inbreeding decline (Kalin Arroyo and Uslar, 1993; Wesselingh and Arnold, 2000; Schrieber et al., 2021). Research on plant breeding systems and pollination characteristics has focused on the breeding systems and pollination characteristics of different species in the same environment or at different altitudes in the same environment (Stucky et al., 2018). However, there is a lack of research on the breeding systems and pollination characteristics of populations of the same plant in different environments, particularly in those with different slope orientations. Therefore, studying the effects of different slope orientations on plant breeding systems and pollination characteristics can elucidate the sexual reproductive characteristics and reproductive strategies of plants in special environments.

**Figure 1:**
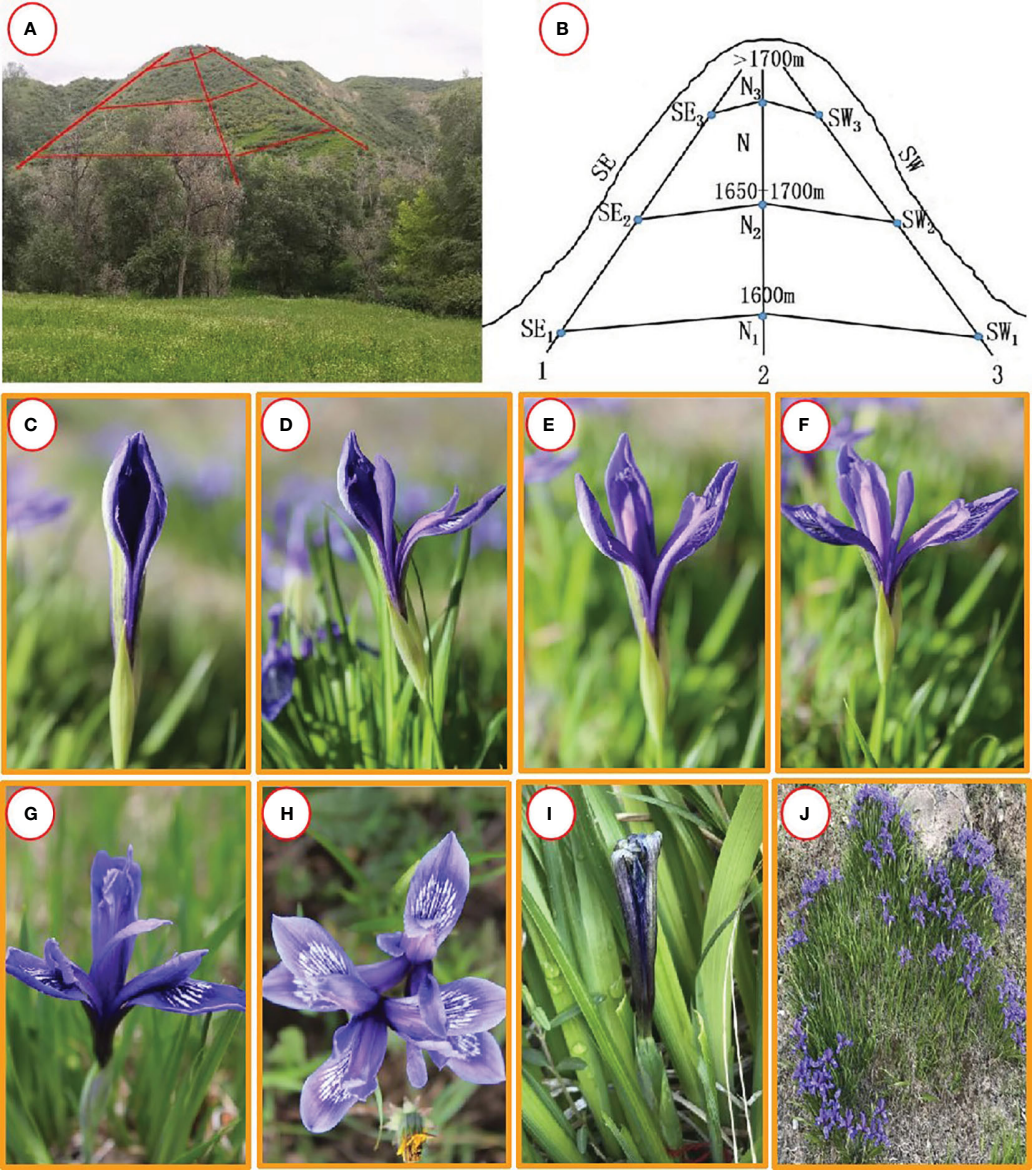
Habitat and opening process of *Iris ruthenica.* **(A, B)** Habitat ; **(C)** Bud; **(D)** Onset of opening; **(E)** Tepals expansion; **(F–H)** Tepals expansion completely; **(I)** Wilting; **(J)** Plant. cluster.

At present, there are approximately 300 species of irises worldwide, mainly distributed in the northern temperate zone (Roguz et al., 2020), and 58 species in China (Zhao, 1985). Among them, *Iris ruthenica* Ker-Gawl. (it sometimes called “Russian iris”) is a perennial plant of the Iridaceae family, which is rampant in grasslands in the degraded mountainous areas of northern Xinjiang, China (Animal Husbandry Department of Xinjiang Uygur Autonomous Region, 1990; Boltenkov and Alexey, 2018). This species is distributed in patches and grows densely in warm and humid environments. It is widely distributed on the shady slopes of the middle mountain belt at an altitude of 1100–2500 m in the Tianshan and Altai Mountains of Xinjiang and on the sunny slopes of the mountain coniferous forest meadow belt, between forest grasslands, in forests, and in forest-edge grasslands (Wang et al., 2021). It is a widely distributed and abundant grassland species in natural grasslands in the central and western sections of the Tianshan Mountains (**Figure 1**).

*Iris ruthenica* possesses cloning and sexual reproduction characteristics and has slender and dense rhizomes, highly developed and strong regenerative ability, and resistance to trampling. Its ability to recover growth and reproduction not only affects the formation of other species but also reduces the quality of grassland, which is an indicator of grassland degradation (Tayier et al., 2020).The plant has poor palatability, and livestock do not feed on it during spring and summer. Usually, it turns green in mid-April and blooms and bears fruit from May to July. The flowering and fruiting characteristics differ among plants on different slopes, and most small flowers bloom according to the size of plant clusters. During the flowering process, insects visit and pollinate the flowers (Abudouwali et al., 2018; Wang et al., 2021). At present, research on this species is limited to its nomenclature history (Boltenkov and Alexey, 2018), seed germination characteristics (Abudouwali et al., 2018), compound content in nutrient organs (Sedelnikova and Kukushkina, 2017), ornamental characteristics and germplasm resources (Liu et al., 2016), introduction and cultivation (Mu and Liang, 2013), and flowering phenology and reproductive characteristics (Wang et al., 2021). No research has investigated breeding systems, pollination characteristics, or strategies for adapting to the environment and ensuring reproduction.

Based on the reproductive and distribution characteristics of this species, the following hypothesis was proposed: as the slope direction changes, population density, flowering phenology, and flower characteristics will differ, and pollinating insects are attracted through larger flower displays to promote outcrossing and ensure the fitness of offspring. Alternatively, under stressful environmental conditions, because of the lack of effective pollinators for the species, it is more likely to achieve fitness benefits through increased self-pollination reproductive strategies. To verify this hypothesis, this study aimed to address the following scientific questions: (1) Is there a difference in the population density of this species on different slopes, and do the flowering phenological parameters of this species, such as flowering time and number, change under different microenvironmental conditions? What is its impact on reproductive success? (2) What are the differences in the flower morphology and breeding systems of this species with different slope orientations? and (3) Does the type, quantity, and frequency of pollinators differ as the slope direction changes?

## 2 Materials and methods

### 2.1 Study species

*Iris ruthenica* is a perennial, rhizomatous, clonal, herbaceous plant belonging to the Iridaceae family, commonly found in degraded mountainous grasslands. Its phenotypic traits include bifurcated rhizomes that extend obliquely, strip-shaped leaves with sheathed bases, a slender flower stem bearing 2–3 stem leaves, two bracts with membranous edges of reddish purple enclosing a single flower, spherical capsules that crack open after maturity, and spherical seeds with milky white appendages. The plant typically turns green in mid-April, flowers, and bears fruit from May to July, and starts to wither and turn yellow from August to September (Abudouwali et al., 2018; Tayier et al., 2020; Wang et al., 2021).

### 2.2 Study area

This study was conducted at the Xiejiagou Grassland Internship Base of Xinjiang Agricultural University in Miaoergou Township, Urumqi County, with geographical coordinates of 87° 37’ 21’’ E; 43° 47’ 07’’ N and an altitude of 1600–1800 m. The region experiences a large temperature difference between day and night and receives a large amount of rainfall with a temperate, continental climate. The annual average temperature is 3.3 °C, with an extreme maximum temperature of 42 °C and an extreme minimum temperature of −41.5 °C. The average annual frost-free period is 179 d. The average annual precipitation is 350–500 mm, and evaporation is 2600 mm (Hasimu et al., 2017).

### 2.3 Research methods

The experiment was conducted from April to July 2018 and 2019, with a selection of different slope orientations of the Xiejiagou grassland microbiota (**Figure 1**); we selected the SE, N, and SW slopes and applied the transect method. On each slope, we marked a transect line following the altitude gradient and selected three sampling points along each transect, with a distance of approximately 40–50 meters between consecutive points. Across the three slopes, there were a total of nine sampling points (**Table 1**, **Figure 1**).

**Table 1:** The population density of *Iris ruthenica* across three slope orientations (mean ± SE).

Population type	Slope orientation	Point	Slope	Altitude	Vegetation type	Light	Vegetation coverage	I. ruthenica coverage	Height of plant (cm)	Number of clusters in the sample	Diameter of plant clusters (cm)	Total number of flowers
Natural population	SE	SE_1−3_	30°	1600–1800	Grassland	Sufficient	53%	35%	15.5 ± 0.4^a^	13 ± 3^b^	99 ± 4^a^	45 ± 5^b^
	N	N_1−3_	33°	1600–1800	Grassland	Weak	90%	74%	19.3 ± 0.8^b^	33 ± 2^a^	70 ± 3^b^	24 ± 2^a^
	SW	SW_1−3_	34°	1600–1800	Grassland	Sufficient	67%	52%	17.8 ± 0.3^b^	27 ± 4^b^	76 ± 5^b^	46 ± 3^b^

Different superscript letters within a column indicate significant differences (P< 0.05).

#### 2.3.1 Investigation of population density of *I. ruthenica* for different slope orientations

During the flowering period of the *I. ruthenica*, a sample survey method was used to select five samples from SE_2_, N_2_, and SW_2_. Within each sampling point, we set up a 5 m × 5 m quadrat and repeated the process three times. We estimated the vegetation coverage and *I. ruthenica* coverage in each quadrat, documented the types of vegetation present, counted the number of clonal clumps of *I. ruthenica*, and measured the diameter and height (height of the flower) of the clonal clumps of *I. ruthenica* within the quadrat.

#### 2.3.2 Flowering phenology of *I. ruthenica* at the population and individual levels for different slope orientations

To understand the changes in the flowering phenology of *I. ruthenica* with changes in slope orientation, 20 clusters of plants with good growth and of roughly the same size before flowering were artificially marked at various points, according to the standard described in Dafni (1992). The total numbers of flowers and flowering clusters were recorded at 09:00 every day. At the individual level, this was expressed as the average of all marked individuals; at the population level, the initial flowering period was when 25% of the individuals were flowering, the peak flowering period was marked by 50% of the individuals flowering, and the final flowering period was when 95% of the plants had finished flowering (Li et al., 2006). Differences in the flowering phenology of *I. ruthenica* on slopes with different orientations were analyzed at the population and individual levels.

#### 2.3.3 Phenology and flowering dynamics of single *I. ruthenica* flowers for different slope orientations

To understand the single-flower flowering process and dynamics of this species, 20 plant clusters were labeled as SE_2_, N_2_, and SW_2_, with each cluster labeled as an unopened flower. The opening and wilting times of the flowers were observed and recorded, and the duration of a single-flower opening was calculated based on the average value of all single flowers. One day was chosen during the peak flowering period, and the number of *I. ruthenica* flowers was counted every 2 h from 09:00 to 19:00.

#### 2.3.4 Flower characteristics of *I. ruthenica* for different slope orientations

To understand the floral characteristics of this species for different slope orientations and during the peak flowering period, 40 plant clusters were selected from SE_2_, N_2_, and SW_2_ with one flower per cluster. The diameter of the corolla, length and width of the perianth, filament, anther, style, and ovary, as well as the distance of the stigma above the anther, were measured using an SF2000 electronic Vernier caliper (accuracy: 0.02 mm).

Twenty plant clusters were randomly marked in SE_2_, N_2_, and SW_2_. A flower bud in each cluster that was developing synchronously and was about to open was selected and bagged. When the flowers were completely open and the anthers began to disperse, the net bag was removed. A 2 μL capillary tube was used to absorb nectar. The length of the nectar was measured, and the volume of the nectar was calculated as 2 μL/capillary length × the length of the nectar in the capillary tube.

Flower buds from SE_2_, N_2_, and SW_2_ labeled plants that were about to open were selected; all anthers were quickly removed using tweezers and placed separately into 1.5 ml centrifuge tubes containing 1 ml of FAA fixative. Samples were transferred to the laboratory for volume determination. The anthers were ground into flocculent form with a glass rod. A pollen suspension was prepared, and a micropipette was used to aspirate 1 μL of the pollen suspension. This was placed on a glass slide, and the number of pollen grains was counted under an optical microscope. Each flower was analyzed six times, and the average number of pollen grains was calculated. The total number of pollen grains per flower was calculated as n × 1000. The ovary of each flower was cut open using a dissecting needle, and the number of ovules was counted.

#### 2.3.5 Detection of breeding systems

We selected 30 plant clusters with roughly equal diameters in SE_2_, N_2_, and SW_2_ and labeled seven flowers in each cluster. The following seven treatments were performed: (1) natural pollination; (2) bagged after emasculation; (3) unbagged after emasculation; (4) bagged without emasculation; (5) geitonogamy; (6) artificial cross-pollination; and (7) artificial self-pollination.

The fruit was collected before it ripened and had not yet cracked; the number of mature fruits, mature seeds, aborted seeds, and unfertilized ovules were counted, and the fruit bearing rate (Equation 1), total number of ovules (Equation 2), seed setting rate (Equation 3), embryo maturation rate (Equation 4), fertilization rate (Equation 5), and pollen limitation (Equation 6) were calculated (Zhang and Tan, 2009; Abdusalam et al., 2012).


(1) $$\begin{array}{c} \mathrm{Fruit bearing rate}\\ \;=\;\left(number\;of\;actual\;fruits\;/\;number\;of\;processed\;flowers\right)\;\times \;100, \end{array}$$

(2) $$\begin{array}{c} Total\;number\;of\;ovules\\ \;=\;number\;of\;mature\;seeds\;+\;number\;of\;aborted\;seeds\;\\ +\;number\;of\;unfertilized\;ovules, \end{array}$$

(3) $$\begin{array}{c} Seed\;setting\;rate\;\left(\%\right)\\ \;=\;\left(number\;of\;mature\;seeds/total\;number\;of\;ovules\right)\;\times \;100, \end{array}$$

(4) $$\begin{array}{c} Embryo\;maturation\;rate\;\left(\%\right)\\ \;=\;[number\;of\;mature\;seeds\;/\;(number\;of\;mature\;seeds\\ \;+\;number\;of\;aborted\;seeds)]\;\times \;100, \end{array}$$

(5) $$\begin{array}{c} Fertilization\;rate\;\left(\%\right)\\ \;=\;[(number\;of\;mature\;seeds\;\;+\;number\;of\;aborted\;seeds)\\ \;/\;total\;number\;of\;ovules]\;\times \;100. \end{array}$$

(6) $$\begin{array}{c} Pollen\;limitation\\ \;=(artificial\;cross-pollination\;seed\;setting\;rate\;-\;self\\ -pollination\;seed\;setting\;rate)\;/\;artificial\;cross\\ -pollination\;seed\;setting\;rate, \end{array}$$

#### 2.3.6 Collection and behavioral observations of flower-visiting insect species

On sunny days, insect nets were used to capture visiting flower insects, which were then placed in bottles of poison to cause death. Some of the specimens were dried for species identification, whereas the others were observed using a Nikon SMZ-1000 digital stereomicroscope to determine whether they carried pollen from *I. ruthenica*.

During the peak flowering period, three plant clusters from various locations were randomly selected and observed on three sunny days (30 min/h). The visited parts, visiting sequence, and residence time (s) of the insects visiting each flower were carefully observed. The number of consecutive flower visits, flight time within plants, and flight time between plants of the visiting insects were tracked and observed while also recording them in detail using video equipment. The frequency of flower visits was expressed as times·flower^−1^·h^−1^.

### 2.4 Data analysis

SPSS 22.0 (SPSS Inc., Chicago, IL, USA) statistical software was used for data processing. The analyzed data underwent normal distribution and homogeneity of variance tests. Data that did not meet the criteria underwent logarithmic or arcsine square root transformations. Significant differences were tested on data that conformed to normal distribution and homogeneity of variance. One-way ANOVA was used to compare significant differences in flowering phenology and floral characteristics among three slope aspects, seed setting rates among seven pollination treatments, and visitation frequencies of different pollinating insects at different times within the same slope aspect. Sigmaplot 10.0 software was used for graphical representation.

## 3 Results

### 3.1 Population density of *I. ruthenica* for different slope orientations

The vegetation coverage of the N, SW, and SE slopes was 90%, 67%, and 53%, respectively, and I. ruthenica coverage was 74%, 52%, and 35%, respectively. Plant height and diameter differed between the SE, N, and SW slopes (P< 0.05), but there were no statistical differences between the N and SW slopes. The number of plant clusters on the N slope was higher than that on the SE and SW slopes (P< 0.05), but the total number of flowers on the N slope was lower than on the SE and SW slopes (P< 0.05). There were no differences between the SE and SW slopes (P > 0.05) (**Table 1**). The distribution of I. ruthenica on the N slope was relatively dense, whereas on the SE and SW slopes, the distribution of I. ruthenica was sparse.

### 3.2 Flowering phenology of *I. ruthenica* at the population and individual levels for different slope orientations

#### 3.2.1 Flowering phenology at the population level

**Figure 2:**
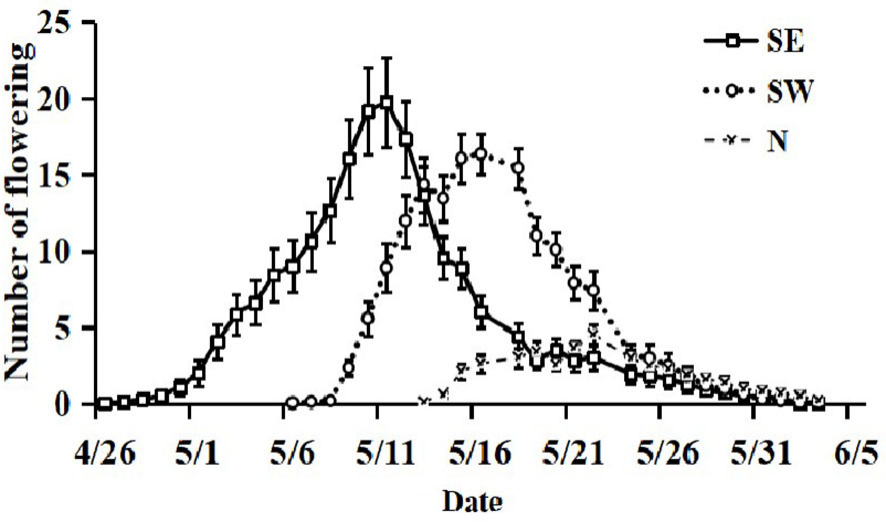
The flowering phenology of *Iris ruthenica* at the population and individual level across three slope orientations. Data are expressed as the mean ± SE.

The SE slope population bloomed the earliest and had a flowering period of up to 30 d, with the SE_2_ population having the earliest initial flowering period; next, the SW slope population began to bloom, with a flowering period of up to 24 d. The N slope population bloomed the latest and generally lasted for 18–19 d. The last flowering date of the populations was consistent (**Figure 2**). The slope direction affected the flowering time and duration of the flowering period of *I. ruthenica*s.

#### 3.2.2 Flowering phenology at the individual level

On the SE slope, SE_2_ had the highest average daily flowering. Each plant cluster had a total of 30 ± 6 flowers on the SE slope. The flowering process was slow from the first 2–3 d and reached the peak flowering period on the 15th day. On the SW slope, SW_3_ had the most flowers on average per day. The total number of flowers per plant cluster on the SW slope was 23 ± 2, and it reached its peak flowering period on the 10th day. On the N slope, the average daily flowering number of the three sampling points was similar, with a maximum of 7 ± 1 flowers per plant cluster, and the maximum number of flowers blooming on a single day was 5 ± 1 (**Figure 2**). Slope direction affected the number of flowers in *I. ruthenica*s.

### 3.3 Flowering phenology and dynamics of single *I. ruthenica* flowers for different slope orientations

#### 3.3.1 Flowering phenology at the single-flower level

The duration of single-flower opening on the SE slope was 5.8 ± 0.17 d, on the SW slope was 5.1 ± 0.12 d, and on the N slope was 3.3 ± 0.1 d. There was a difference in the duration of single-flower blooming between the N, SE, and SW slopes (P< 0.05), whereas there was no significant difference between the SE and SW slopes (P > 0.05). Therefore, slope direction affected the duration of single-flower blooming in *I. ruthenica*.

**Figure 3:**
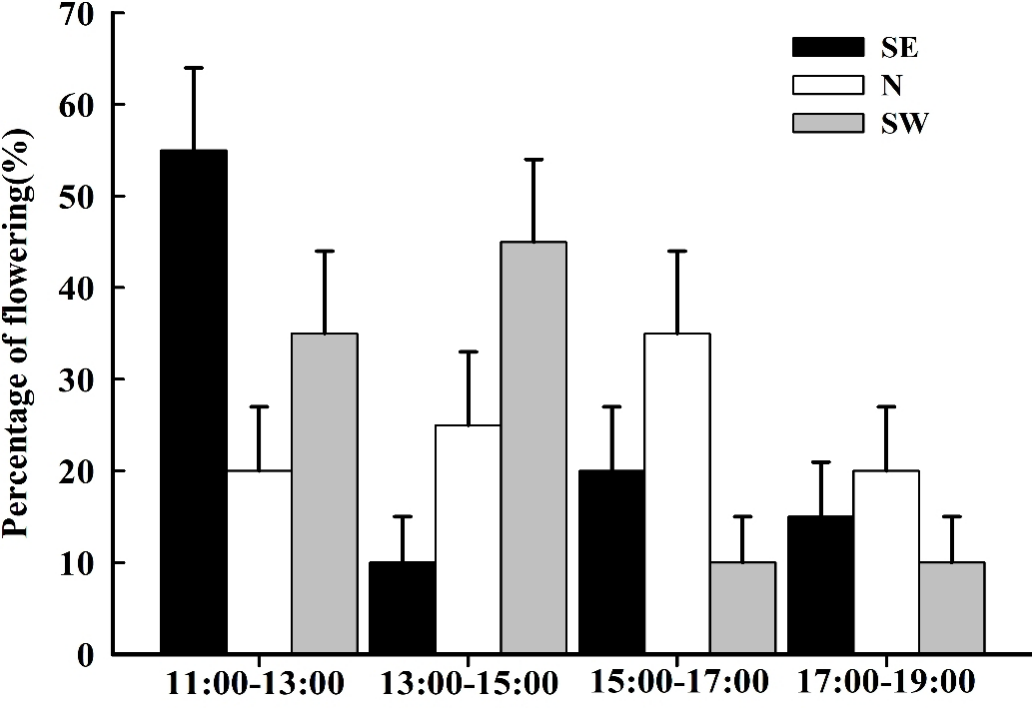
The flowering dynamics of *Iris ruthenica* across three slope orientations . Data are expressed as the mean ± SE.

The blooming of *I. ruthenica* lasted from 11:00–19:00. The flowering peak on the SE slope was from 11:00–13:00, that on the SW slope was from 13:00–15:00, and that on the N slope was from 15:00–17:00 (**Figure 3**).

#### 3.3.2 Blooming dynamics

When a flower bloomed, the outer petals of the perianth slowly opened, usually in sunny weather with higher temperatures or under the influence of wind. In rainy weather at lower temperatures, the petals opened slowly. After the perianth petals unfolded, the anthers began to crack and scatter pollen, and pollinators began to move frequently. The style was petal-shaped and folded downwards, the stamens were tightly attached to the outer side of the petalated style, and the relative positions of the female and male stamens remained unchanged. When the flower wilted, the inner perianth first compressed toward the center of the corolla, followed by the style, filaments, and anthers moving toward the center. The outer perianth also contracted and gradually twisted and tangled together, while the perianth gradually faded and eventually withered (**Figure 1**).

### 3.4 Flower characteristics of *I. ruthenica* for different slope orientations

The distance between the stigma and the anther differed among slope orientations (F = 14.64, P< 0.05), with SE > SW > N. Felly tepals length (F = 0.66, P = 0.52), Felly tepals width (F = 5.23, P< 0.05), inner tepals length (F = 2.73, P = 0.07), inner tepals width (F = 1.71, P = 0.19), filament length (F = 2.11, P = 0.13), ovary width (F = 3.04, P = 0.05), and nectar content (F = 2.01, P = 0.14) did not differ. The diameter of the corolla (F =17.68, P< 0.05) and the number of ovules (F = 13.95, P< 0.05) differed between the N slope and the SE and SW slopes, but those of the SE and SW slopes were similar. Anther length (F = 19.11, P< 0.05), ovary length (F = 7.41, P< 0.05), and style length (F = 6.04, P< 0.05) differed between the N and SE slopes. There was a difference in the number of pollen grains between the N and the SW slopes (F = 2.89, P = 0.06; **Table 2**).

**Table 2:** The flower characteristics of *Iris ruthenica* across three slope orientations (mean ± SE).

Slope orientation		SE	N	SW	F	P
Corolla diameter (mm)		58.86 ± 1.02^a^	50.94 ± 0.96^b^	56.78 ± 0.94^a^	17.68	< 0.05
Felly tepals	Length (mm)	53.23 ± 0.58^a^	53.63 ± 0.68^a^	54.24 ± 0.61^a^	0.66	0.52
	Width (mm)	12.42 ± 0.18^a^	13.33 ± 0.27^a^	13.20 ± 0.18^a^	5.23	< 0.05
Inner tepals	Length (mm)	50.56 ± 0.48^a^	49.96 ± 0.61^a^	51.80 ± 0.60^a^	2.73	0.07
	Width (mm)	5.39 ± 0.12^a^	5.08 ± 0.12^a^	5.21 ± 0.11^a^	1.71	0.19
Stamen	Length of filaments (mm)	23.24 ± 0.33^a^	22.08 ± 0.57^a^	22.96 ± 0.28^a^	2.11	0.13
	Length of anthers (mm)	9.86 ± 0.15^b^	9.57 ± 0.17^a^	10.97 ± 0.18^ab^	19.11	< 0.05
Pistil	Ovary length (mm)	9.18 ± 0.19^a^	8.14 ± 0.18^b^	8.64 ± 0.20^ab^	7.41	< 0.05
	Ovary width (mm)	3.64 ± 0.05^a^	3.44 ± 0.07^a^	3.64 ± 0.07^a^	3.04	0.05
	Length of style (mm)	37.08 ± 0.41^a^	34.27 ± 0.79^b^	36.07 ± 0.46^ab^	6.04	< 0.05
Stigma above anther (mm)		4.03 ± 0.25^a^	2.55 ± 0.22^b^	2.74 ± 0.23^c^	14.64	< 0.05
Number of pollen grains		26203 ± 1206^ab^	23256 ± 895^a^	26886 ± 1268^b^	2.89	0.06
Number of the ovules		67 ± 2^a^	52 ± 3^b^	68 ± 2^a^	13.95	< 0.05
Nectar volume (µL)		0.88 ± 0.09^a^	1.05 ± 0.08^a^	0.83 ± 0.07^a^	2.01	0.14

Different superscript letters within a row indicate significant differences (P< 0.05);

### 3.5 Breeding system

The bagged seed setting rate after emasculation was 0%. The seed-setting rate of specimens bagged without emasculation on the SE slope was 6.33%, whereas the seed-setting rate on the N and SW slopes was 0%. There was no difference (P > 0.05) between natural pollination and unbagging after emasculation. Seed-setting rate did not differ between artificial self-pollination and artificial cross-pollination (P > 0.05). Pollen limitation values of the SE, N, and SW slope were 0.35, 0.12, and 0.34, respectively, with no differences (P > 0.05) among natural pollination, artificial cross-pollination, and self-pollination (**Table 3**).

**Table 3:** The seed-setting rate of *Iris ruthenica* under different pollination treatments across three slope orientations (mean ± SE).

Pollination treatments	*n*	Seed set (%)		
		SE	N	SW
Natural pollination	30	40.00 ± 2.75^bc^	32.34 ± 6.41^a^	49.43 ± 5.28^b^
Bagged after emasculation	30	0	0	0
Unbagged after emasculation	30	35.71 ± 3.12^bc^	30.20 ± 4.81^a^	35.30 ± 3.97^ab^
Bagged without emasculation	30	6.33 ± 0.51^d^	0	0
Geitonogamy	30	35.33 ± 1.75^bc^	30.20 ± 4.81^a^	35.52 ± 1.37^ab^
artificial cross-pollination	30	37.34 ± 1.48^bc^	31.86 ± 5.09^a^	41.52 ± 2.52^b^
artificial self-pollination	30	24.14 ± 1.44^c^	27.74 ± 7.30^a^	27.32 ± 4.89^b^

Different superscript letters within a column indicate significant differences (P< 0.05); n indicates the number of samples.

### 3.6 Types and behaviors of flower-visiting insects

**Figure 4:**
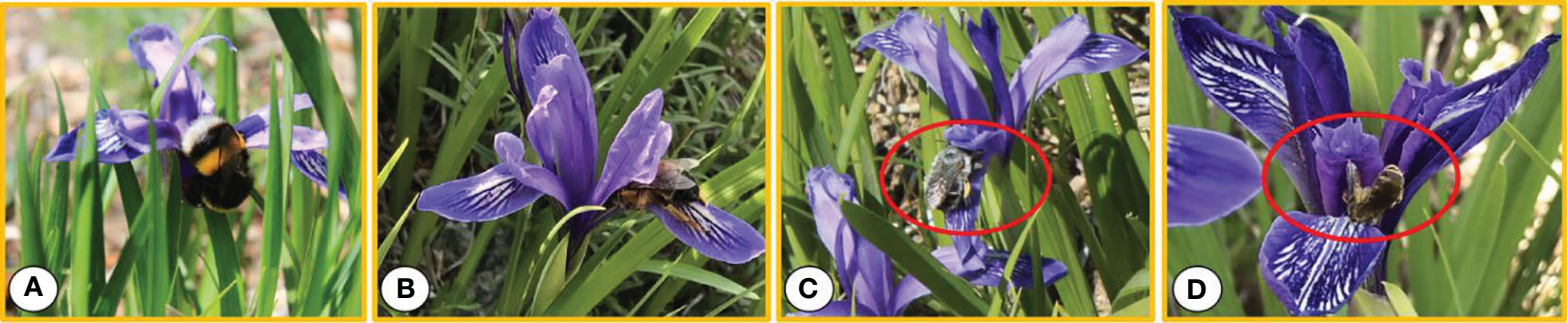
Pollinators of Iris ruthenica. **(A)** Bombus sp.; **(B)** Amegilla leptocoma; **(C)** Andrena sp.; **(D)** Halictus sp.

Four types of insects visited the flowers of *I. ruthenica*, namely bees, ants, flies, and mosquitoes (Hymenoptera and Diptera). Flies, ants, and mosquitoes were the least frequent. *Bombus* sp., *Amegilla leptocoma*, *Andrena* sp., and *Halictus* sp. were the main pollinating insects (**Figures 4A–D**). *Bombus* sp. were relatively large and crawled along the outer perianth segments to the base of the perianth tube to suck nectar. When they burrowed between the perianth segments and the style, the chest and abdomen made contact with the anther, collecting pollen (**Figure 4A**). The individual *Amegilla leptocoma* were smaller than the bear bees. They continuously visited the flowers, crawled slowly between the perianth and style toward the base of the flowers, extended the mouthpiece to suck nectar, and the pollen was attached at this time. When they climbed out of the channel, their bodies came into contact with the stigma (**Figure 4B**). *Andrena* sp., with long proboscises, burrowed into the pollination channels and fed on nectar deep in the flower base (**Figure 4C**). *Halictus* sp. had the smallest body size. Some remained on the outer flower cover for a moment before flying away, whereas others slowly burrowed into the channel along the edge of the flower cover or fell directly behind it, crawling back and forth to feed on pollen. Pollen was attached to their antennae and body surfaces (**Figure 4D**). *Xylocopa* sp. visited and were the most active, but they did not pollinate the flowers.

### 3.7 Visiting frequency of pollinating insects for different slope orientations

**Figure 5:**
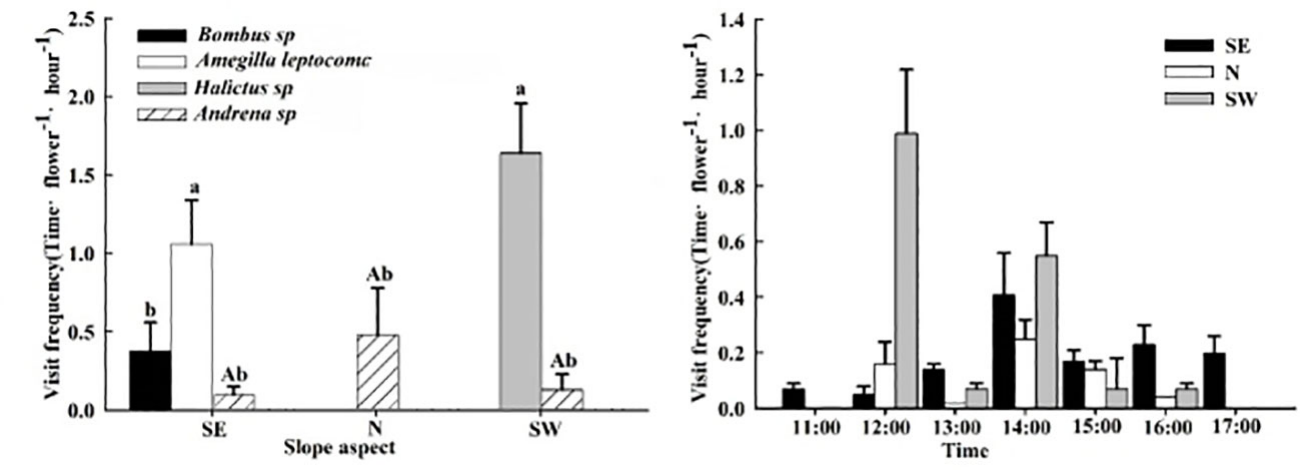
The frequency and dynamics of pollinating insect visits to *Iris ruthenica* flowers across three slope orientations. Data are expressed as the mean ± SE. Different uppercase letters indicate significant differences in the frequency of flower visits by the same pollinator on different slopes, and different lowercase letters indicate significant differences in flower visitation rates for the same flowers by different pollinators and in the frequency of flower visits by different pollinators on the same slope (P < 0.05).

The pollinating insects on the SE slope were *Bombus* sp., *Amegilla leptocoma*, and *Andrena* sp., those on the N slope were *Andrena* sp., and those on the SW slope were *Andrena* sp. and *Halictus* sp. There were no differences in the frequency of *Andrena* sp. visiting the flowers between the different slope orientations (P > 0.05). On the SE slope, the frequency of flower visits by the *Amegilla leptocoma* was higher than that of the *Bombus* sp. and *Bombus* sp. (P< 0.05), at 1.06 ± 0.28 times·flower^−1^·h^−1^, with a single-flower residence time of 7.05 ± 0.19 s. Each insect could consecutively visit 5.95 ± 0.69 flowers within the plant cluster (**Table 4**), indicating that the main pollinating insect on the SE slope was the *Amegilla leptocoma*. On the N slope, the frequency of flower visits by *Bombus* sp. was 0.48 ± 0.30 times·flower^−1^·h^−1^, and the residence time on a single flower was 2.58 ± 1.27 s. Each insect visited 3.22 ± 0.45 flowers, indicating that the main pollinating insect on the N slope was the *Bombus* sp. The frequency of flower visits by the *Halictus* sp. on the SW slope was higher than that of the *Bombus* sp. (P< 0.05), at 1.64 ± 0.62 times·flower^−1^·h^−1^. The longest single-flower residence time was 44.38 ± 7.57 s and 3.09 ± 0.43 flowers could be visited consecutively, indicating that the main pollinating insect on the SW slope was the *Halictus* sp. (**Figure 5**; **Table 4**).

**Table 4:** The flower visitation behavior of pollinating insects across three slope orientations (mean ± SE).

Observation item	SE			N	SW	
	*Bombus* sp.	*Amegilla leptocoma*	*Andrena* sp.	*Andrena* sp.	*Halictus* sp.	*Andrena* sp.
Residence time (s)	5.87 ± 0.19	7.05 ± 0.19	2.97 ± 1.50	2.58 ± 1.27	44.38 ± 7.57	7.07 ± 0.36
Number of continuous visits	3.83 ± 0.17	5.95 ± 0.69	2.27 ± 1.13	3.22 ± 0.45	3.09 ± 0.43	4.33 ± 1.59
Flight time between flowers (s)	1.22 ± 0.14	1.84 ± 0.92	1.76 ± 0.88	3.03 ± 0.23	2.42 ± 0.30	2.74 ± 0.38
Flight time between plants (s)	0.55 ± 0.10	5.33 ± 2.69	–	–	–	2.21 ± 0.27

The daily activity of pollinating insects on the SE slope was from 11:00–18:00, and the peak flower-visiting period was from 14:00–15:00. For the N slope and SW slope pollinators, the daily activity time was 12:00–17:00 and the peak periods for visiting flowers were 12:00–13:00 and 14:00–15:00, respectively (**Figure 5**).

## 4 Discussion

The flowering time, flowering duration, and final flowering time of plants are parameters that quantify the flowering phenology of a species. Flowering phenology at the community level is caused by both exogenous (biotic and abiotic) and endogenous (genetic and phylogenetic) factors (Van Schaik et al., 1993; Linder, 2020). Slope orientation is an important terrain factor that causes differences in solar radiation intensity, resulting in differences in the number and timing of plant flowering, thereby affecting the frequency of pollinator visits and final fruit and seed yields (Dafni, 1992; Olliff-Yang and Ackerly, 2020; Nagai et al., 2022).

The current study found that the SE slope bloomed the earliest, with a longer duration of group and single flowering periods. Other plants associated with the iris had not entered the flowering period; a large number of flowers together have a visual appeal for pollinators, which is beneficial for plants to obtain more mating opportunities (Steinacher and Wagner, 2010; Guo et al., 2023). The total number of flowers in the SE slope plant clusters was the highest, and the flowers on the same plant cluster bloomed in turn, allowing each plant cluster to have the highest number of flowers during the entire flowering period, which is conducive to attracting pollinating insects. The flowering times of the SW and SE slopes were consistent, with a substantial display of flowers during the peak flowering period, which has a considerable advertising effect. This attracts pollinators, and opening a large number of flowers can increase the pollen supply and improve individual male fitness, which is a strategy adopted to achieve successful reproduction (Xiao et al., 2004). However, the N slope was exactly the opposite, with the latest flowering period, small clusters, few flowers, and small flower displays, resulting in a weak attraction to pollinators. This suggests that the N-slope population might face more substantial pollination restrictions. Despite no differences in fertilization rates on different slopes (P > 0.05), the seed setting rate and embryo maturation rate of the N slope were lower than those of the SE and SW slopes under relatively more severe environmental conditions (P< 0.05), which may be due to resource limitations on the production of *I. ruthenica* seeds on the N slope. The differences observed in this species growing in the same environment are most likely due to differences in light intensity, light duration, soil temperature, soil humidity, and soil moisture caused by slope orientation, in addition to genetic characteristics. These factors can limit the resources that plants obtain from the environment, leading to differences in the distribution, flowering, and fruiting characteristics of the species on different slopes.

Self-pollination, cross-pollination, and mixed mating of plants are influenced not only by factors such as floral characteristics, flowering duration, pollinator behavior and activity, and population structure (Abdusalam et al., 2012) but also by climatic conditions such as habitat and geographical distribution differences (Castro et al., 2008). A common hypothesis for the reproductive security of alpine plants is that, as environmental and climatic conditions become more severe, the limitation of pollinators forces plants to increase their self-pollination ability as a reproductive guarantee against harsh environmental conditions that restrict insect access to flowers, to ensure ovule fertilization and seed production (Hillyer and Silman, 2010; Totland and Schulte-Herbrüggen, 2003; Inouye, 2020). The current study found that the corolla diameter, distance between the stigma and anther, anther length, ovary length, number of pollen grains, and number of ovules of the N slope *I. ruthenica*s were smaller than those of the SE and SW slope flowers (P< 0.05). The seed setting rates under the same pollination treatments were similar (P > 0.05), except for natural pollination (P< 0.05). After emasculation, bagging did not produce seeds, indicating that the species cannot undergo apomixis. Regarding the bagged without emasculation treatments, neither the N nor the SW slope flowers bore seeds, and the SE slope flowers had a low seed-bearing rate, which may have been due to the penetration of small insects such as ants and thrips. This indicates that this species does not have autonomous self-pollination.

There was no difference (P > 0.05) between natural pollination and the unbagged after emasculation treatment, indicating that the sturdiness of this species relies on pollination by pollinators and that there is a certain degree of self-pollination. There was no significant difference in the seed setting rate between artificial self-pollination and artificial heterozygous cross-pollination (P > 0.05), indicating that the species is self-compatible. The values from the N slope for these two treatments were small, indicating a high degree of self-compatibility. There was no difference (P > 0.05) between natural pollination, artificial cross-pollination, and self-pollination on the different slopes, indicating that this species can reproduce sexually through self- and cross-pollination. The plant has a mixed mating system, and the adaptability of self-pollinated seeds was lower than that of cross-pollinated seeds. Under the comprehensive effect of environmental factors, the N slope had a smaller flower display but a higher degree of self-compatibility, ensuring a specific seed yield under stressful resource and environmental conditions. The SE and SW slopes attracted more pollinating insects through larger flower displays, thereby ensuring successful reproduction.

Attracting pollinators through the color and size of flowers is a vital component of plant reproductive fitness (Fishbein and Venable, 1996; Ma et al., 2006; Bauer et al., 2017), and the frequency of pollinator visits to insect-pollinated plants is closely related to plant fitness (Potts et al., 2010). Climatic conditions in high-altitude areas are variable, and the types and quantities of pollinating insects vary (Zhang and Tan, 2009; Kudo, 2022). A study on the pollination biology of *Impatiens hainanensis* at different altitudes revealed that the main pollinating insects in low- and medium-altitude populations were the *Amegilla leptocoma*, whereas *Amegillazonata* were the primary pollinators at high altitudes. This may be because *Amegillazonata* were better able to adapt to the harsh environmental conditions at high altitudes (Zhong et al., 2014).

The current study found that the floral structure of *I. ruthenica* was adapted to bee pollination, with the main pollinating insects being *Bombus* sp., *Amegilla leptocoma*, *Andrena* sp., and *Halictus* sp. The color of the flowers, the nectar secreted, and the pollen provided are important rewards for insect pollination (Zhu et al., 2017), and the Felly tepals (hanging petals) provide footholds for pollinators. The inner petal (petal) has a certain visual appeal to pollinators. Different slope orientations resulted in different types and numbers of pollinating insects. The SE and SW slopes had more types and numbers of pollinating insects, whereas the N slope had fewer types and numbers of pollinators and a lower frequency of flower visits under the more stressful environmental conditions. Although there was not much difference in the number of flowers in the plant clusters visited by pollinators in the three habitats, the number of flowers that opened simultaneously in N slope plants was small, which increased cross-pollination within the same plant, resulting in a higher self-pollination rate and leading to an inbreeding decline. The larger flowers on the SE and SW slopes increased the chances of pollinators visiting and pollinating the same or other plant clusters. However, pollinators only visited 3–5 flowers consecutively in the plant cluster, and their self-compatibility was low. Larger flower displays are attractive to pollinators, and the likelihood of cross-pollination within the same plant is low. The SE and SW slopes attracted more pollinators by early flowering and opening a large number of flowers, promoting opportunities for cross-pollination.

In summary, slope orientation had an impact on the population density, flowering phenology, floral characteristics, breeding systems, and pollination characteristics of *I. ruthenica*. The *I. ruthenica* on the SE and SW slopes was distributed in a low and sparse pattern, with a higher total number of flowers in the plant cluster, earlier flowering time, greater flowering quantity, longer duration of flowering, and fewer neighboring species. This, to some extent, promoted the activity and range of insects, ensuring the successful reproduction of this species.

The distribution of *I. ruthenica* on the N slope was relatively dense, but the total number of flowers in each plant cluster was relatively small, the flowering time was later, and the number of flowers was small. There were also many neighboring species, and the number of pollinating insects was relatively small. Increasing the degree of self-compatibility was adopted to compensate for these shortcomings in cross-pollination and ensure successful reproduction in stressful environments.

## Data availability statement

The raw data supporting the conclusions of this article will be made available by the authors, without undue reservation.

## Author contributions

GT: Investigation, Software, Writing – original draft. DH: Conceptualization, Data curation, Methodology, Writing – original draft, Writing – review & editing. TA: Data curation, Supervision, Validation, Writing – review & editing. AY: Conceptualization, Funding acquisition, Project administration, Resources, Supervision, Writing – review & editing.
